# Impact of COVID-19 on the Healthcare of Patients With Inflammatory Bowel Disease: A Comparison Between Epicenter vs. Non-epicenter Areas

**DOI:** 10.3389/fmed.2020.576891

**Published:** 2020-11-30

**Authors:** Yun Qiu, Ying-Fan Zhang, Liang-Ru Zhu, Jin-Shen He, Jin-Yu Tan, Nian-Di Tan, Si-Nan Lin, Xiao-Qing Lin, Subrata Ghosh, Min-Hu Chen, Ren Mao

**Affiliations:** ^1^Department of Gastroenterology, The First Affiliated Hospital of Sun Yat-sen University, Guangzhou, China; ^2^Division of Gastroenterology, Union Hospital, Tongji Medical College, Huazhong University of Science and Technology, Wuhan, China; ^3^National Institute for Health Research Biomedical Research Institute, Institute of Translational Medicine, University of Birmingham and University Hospitals Birmingham Nightingale Hospital (NHS) Foundation Trust, Birmingham, United Kingdom

**Keywords:** COVID-19, inflammatory bowel disease, medical care, telemedicine, epicenter, non-epicenter

## Abstract

**Background and Aims:** The COVID-19 pandemic poses a great challenge to healthcare. We aimed to investigate the impact of COVID-19 on the healthcare of patients with inflammatory bowel disease (IBD) in epicenter and non-epicenter areas.

**Methods:** Patients with IBD from Hubei province (the epicenter of COVID-19) and Guangdong province (a non-epicenter area), China were surveyed during the pandemic. The questionnaire included change of medications (steroids, immunomodulators, and biologics), procedures (lab tests, endoscopy, and elective surgery), and healthcare mode (standard healthcare vs. telemedicine) during 1 month before and after the outbreak of COVID-19.

**Results:** In total, 324 IBD patients from Guangdong province (non-epicenter) and 149 from Hubei province (epicenter) completed the questionnaire with comparable demographic characteristics. Compared to patients in Guangdong province (non-epicenter), significantly more patients in Hubei (epicenter) had delayed lab tests/endoscopy procedures [61.1% (91/149) vs. 25.3% (82/324), *p* < 0.001], drug withdrawal [28.6% (43/149) vs. 9.3% (30/324), *p* < 0.001], delayed biologics infusions [60.4% (90/149) vs. 19.1% (62/324), *p* < 0.001], and postponed elective surgery [16.1% (24/149) vs. 3.7% (12/324), *p* < 0.001]. There was an increased use of telemedicine after the outbreak compared to before the outbreak in Hubei province [38.9% (58/149) vs. 15.4% (23/149), *p* < 0.001], while such a significant increase was not observed in Guangdong province [21.9% (71/324) vs. 18.8% (61/324), *p* = 0.38]. Approximately two-thirds of IBD patients from both sites agreed that telemedicine should be increasingly used in future medical care.

**Conclusions:** Our patient-based survey study in a real-world setting showed that COVID-19 resulted in a great impact on the healthcare of patients with IBD, and such an impact was more obvious in the epicenter compared to the non-epicenter area of COVID-19. Telemedicine offers a good solution to counteract the challenges in an unprecedented situation such as COVID-19.

## Introduction

The pandemic of COVID-19 has tremendously impacted the entire world. This pandemic poses a great challenge to the healthcare of patients with many chronic diseases including inflammatory bowel disease (IBD). It is now clear that IBD is increasing worldwide and has become a global emergence disease in industrial-urbanized societies (1). Characterized by a relapsing and remitting course, patients with IBD need close monitoring and therapy adjustment in order to avoid acute flares. Thus, optimal management of IBD patients requires large healthcare resource utilization (2) which becomes a big challenge for hospitals, especially in the epicenter of disease, who are completely occupied by critical COVID-19 patients and have no room for “general” patients.

Telemedicine might be a virtual solution to counteract such a challenge. In two recently published articles (3, 4), the influence of COVID-19 on the treatment of immune-mediated inflammatory diseases was reported, and telemedicine was proposed as a solution to counteract challenges in healthcare delivery posed by COVID-19. However, the impact of COVID-19 on the healthcare of patients with such diseases, and the role of telemedicine in such a situation has seldom been investigated in a real-world setting.

The aim of this study was to investigate the impact of COVID-19 on the healthcare of patients with IBD using a patient-based survey, and to compare the data before and after the outbreak of COVID-19, both in Hubei province (epidemic) and Guangdong province (non-epidemic) in China.

## Methods

### Survey Design

Electronic questionnaire surveys were carried out to compare IBD patients in Hubei province (epicenter of COVID-19) and Guangdong province (non-epicenter), China. The questionnaire was focused on the change of medications (steroids, immunomodulators, and biologics), procedures (lab tests, endoscopy, and elective surgery), and healthcare mode (standard healthcare vs. telemedicine) during 1 month before and after the outbreak of COVID-19. We also investigated the impact of COVID-19 on attitudes of patients toward telemedicine.

All questions were closed with multiple choice answers. The Chinese questionnaire (Supplementary Material 1) was piloted for comprehensibility among 10 patient-volunteers from the center of Guangzhou.

### Statistical Analysis

Answers were summarized based on the total number of respondents to each question, and missing data for a question were excluded from that particular analysis. Categorical variables were expressed in frequencies and percentages. Continuous variables were expressed as mean and standard deviation (SD) or median and range. Two independent samples were tested by the Student T-test; the analysis of variance or Kruskal-Wallis rank-sum test was used for comparison between multiple groups. The χ^2^ test was performed to compare count data, and a 2-tailed value of *P* < 0.05 was considered statistically significant.

Statistical analysis was performed using GraphPad Prism (5.03, GraphPad Soft- ware, Inc., San Diego, USA).

## Results

In total, 324 IBD patients from Guangdong province (non-epicenter) and 149 from Hubei province (epicenter) completed the questionnaire, and the demographic characteristics were comparable between patients from these two provinces (Table 1).

**Table 1:** The baseline of survey IBD patients from Guangdong and Hubei.

	**Guangdong**	**Hubei**	***P***
	**(*n* = 324)**	**(*n* = 149)**	
Diagnosis			
CD:UC:IBD-U	235:75:14	94:48:7	0.102
Gender			
M:F	207:117	88:61	0.314
Age, *n* (%)			0.043
<16 y	14 (4.3)	4 (2.7)	
16–40 y	217 (67)	93 (62.4)	
>40 y	93 (28.7)	49 (32.9)	
>65 y	0	3 (2)	
Disease duration, *n* (%)			<0.001
≤ 2 y	66 (20.4)	48 (32.2)	
2–5 y	106 (32.7)	68 (45.6)	
5–10 y	92 (28.4)	27 (18.1)	
>10 y	60 (18.5)	6 (4)	

*IFX, infliximab; ADA, adalimumab; MTX, methotrexate; SASP, salazosulfapyridine; 5-ASA, 5-aminosalicylic acid; CD, Crohn's disease; UC, ulcerative colitis; IBD-U, inflammatory bowel disease unclassified; M, male; F, female; y, year*.

### Change in Medications and Procedures During the COVID-19 Outbreak

**Figure 1:**
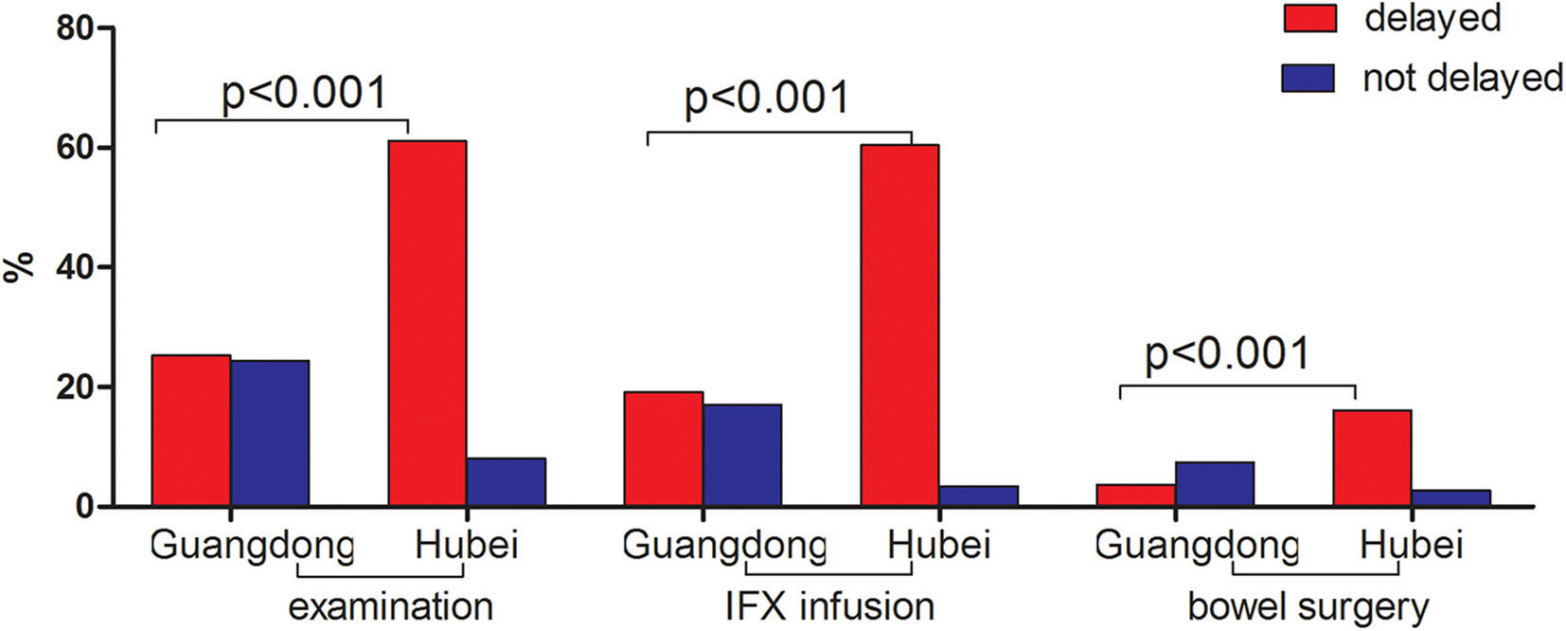
Comparison of medications and procedures between pre-and post-pandemic in Guangdong (non-epicenter) and Hubei (epicenter) province.

**Figure 2:**
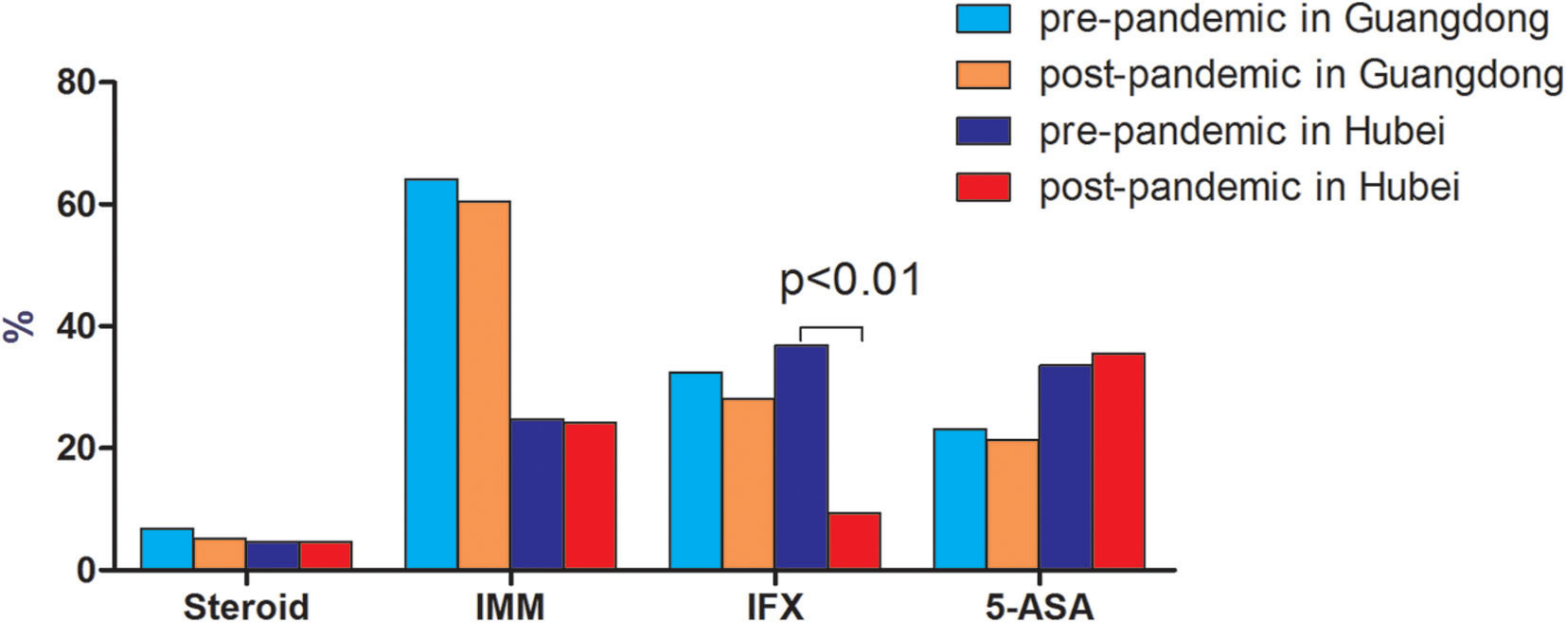
Comparison of medication use between pre-and post-pandemic in Guangdong (non-epicenter) and Hubei (epicenter) province.

Compared to patients in Guangdong province (non-epicenter), significantly more patients in Hubei (epicenter) had delayed lab tests/endoscopy procedures [61.1% (91/149) vs. 25.3% (82/324), *p* < 0.001], drug withdrawal [28.6% (43/149) vs. 9.3% (30/324), *p* < 0.001], and postponed elective surgery [16.1% (24/149) vs. 3.7% (12/324), *p* < 0.001] (Figure 1). There was no significant change in use of steroids, thiopurines, and aminosalicylates before and after the pandemic outbreak in both areas. However, there were significantly more patients with delayed biologics infusions in the epicenter compared to the non-epicenter area [60.4% (90/149) vs. 19.1% (62/324), *p* < 0.001, Figure 2].

### Change in the Healthcare Mode Before and After the Outbreak of COVID-19

**Figure 3:**
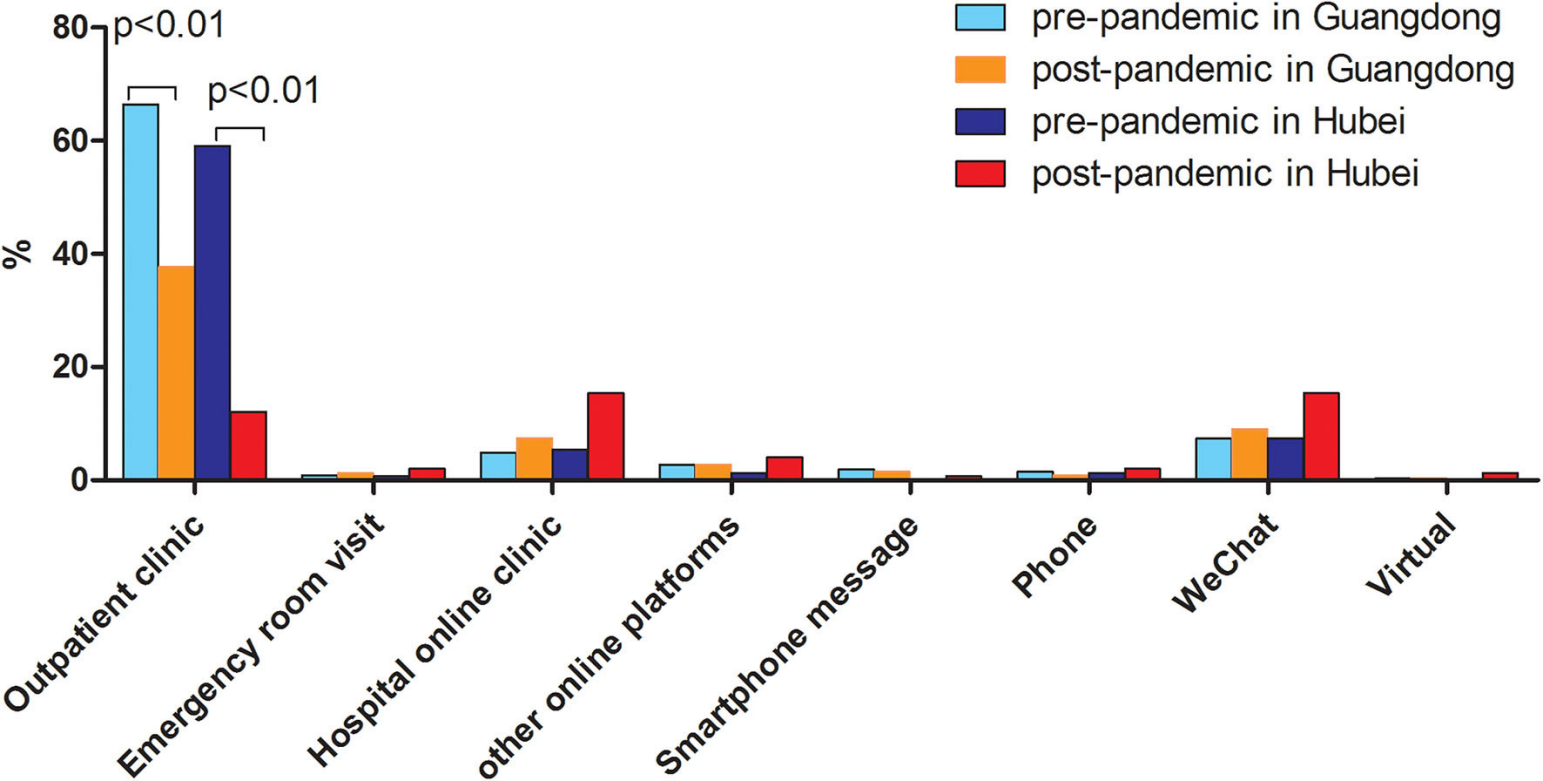
Comparison of mode of medical care between pre-and post-pandemic in Guangdong (non-epicenter) and Hubei (epicenter) province.

The outbreak of COVID-19 resulted in a substantial decrease of patients participating in standard face-to-face visits. The number of patients who attended standard face-to-face visits reduced more dramatically in Hubei province [59.1% (88/149) vs.12.1% (18/149), *p* < 0.001] than that in Guangdong province [66.4% (215/324) vs. 37.7% (124/324), *p* < 0.001] (Figure 3). There was an increased use of telemedicine after the outbreak compared to before the outbreak in Hubei province [38.9% (58/149) vs.15.4% (23/149), *p* < 0.001], while such a significant increase was not observed in Guangdong province [21.9% (71/324) vs. 18.8% (61/324), *p* = 0.38]. Regarding the frequency of telemedicine use, there was a trend toward, though not significantly, a higher percentage of patients using telemedicine (≥3 times) in Hubei province (26/149, 17.5%) compared to that in Guangdong province (39/324, 12.1%) (*p* = 0.082) (Table 2). Among all kinds of telemedicine, hospital-based online clinics and WeChat consultations were the two most used both in Guangdong province and Hubei province. Approximately two-thirds of IBD patients from both sites agreed that telemedicine should be increasingly used in future medical care (Table 2).

**Table 2:** Comparison of use of medication, source to get medication, and way of seeking medical care in Guangdong and Hubei pre- and post-pandemic.

	**Guangdong**		***P***	**Hubei**		***P***
	**(*n* = 324)**			**(*n* = 149)**		
Medication, *n* (%)	Pre	Post	0.118	Pre	Post	<0.001
Steroids	22	17		7	7	
Thiopurine	128	122		28	28	
Thalidomide	61	57		8	6	
Oral MTX	6	7		1	2	
MTX im	13	10		0	0	
IFX	105	91		55	14	
ADA	0	0		1	0	
On trials	1	0		1	0	
SASP	5	3		4	4	
5-ASA	70	66		46	49	
None	10	24		13	42	
Access to medications, *n* (%)	Pre	Post	<0.001	Pre	Post	<0.001
Outpatient clinic	200 (61.7)	124 (38.3)		60 (40.3)	16 (10.7)	
Emergency	6 (1.9)	3 (0.9)		3 (2)	1 (0.7)	
Pharmacy	40 (12.3)	30 (9.3)		42 (28.2)	35 (23.5)	
Online	76 (23.5)	113 (34.9)		26 (17.4)	35 (23.5)	
Way of seeking medical care, *n* (%)	Pre	Post	<0.001	Pre	Post	<0.001
Outpatient clinic	215 (66.4)	122 (37.7)		88 (59.1)	18 (12.1)	
Emergency	3 (0.9)	4 (1.2)		1 (0.7)	3 (2)	
Hospital online clinic	16 (4.9)	24 (7.4)		8 (5.4)	23 (15.4)	
Other online platform	9 (2.8)	9 (2.8)		2 (1.3)	6 (4)	
Message	6 (1.9)	5 (1.5)		0 (0)	1 (0.7)	
Phone	5 (1.5)	3 (0.9)		2 (1.3)	3 (2)	
WeChat	24 (7.4)	29 (9)		11 (7.4)	23 (15.4)	
Video	1 (0.3)	1 (0.3)		0 (0)	2 (1.3)	
No visit	74 (22.8)	150 (46.3)		42 (28.2)	89 (59.7)	

*IFX, infliximab; ADA, adalimumab; MTX, methotrexate; SASP, salazosulfapyridine; 5-ASA, 5-aminosalicylic acid; CD, Crohn's disease; UC, ulcerative colitis; IBD-U, inflammatory bowel disease unclassified; M, male; F, female; y, year*.

## Discussion

The current unprecedented pandemic poses a great challenge to public health resource as well as patients with IBD. A lot of focus has been put on the outcomes of IBD patients with COVID-19. However, attention should be also paid to the impact of COVID-19 on regular IBD patients (non COVID-19 infection) who are the majority of the IBD population. Indeed, a recent survey of members of the European Crohn's and Colitis Organization (ECCO) showed that COVID-19 has disrupted and revolutionized the management of IBD patients, forcing physicians to face new problems (5). The present study using a patient-based survey explored the difference in the impact of COVID-19 on the medical care of IBD patients in the epidemic compared to the non-epidemic area.

As demonstrated in our survey, patients both in the epicenter and non-epicenter areas had limited access to healthcare evident by the decreased number of standard fact-to-fact visits, and delayed examinations, biologics infusion, and selective surgery. The situation was more serious in the epidemic area of Hubei province due to the lockdown of the whole province and the fact that many gastroenterologists were reassigned and directly involved in the care of COVID-19 patients.

Not only is providing adequate follow-up for IBD complicated during the COVID-19 outbreak, but ensuring adequate care of patients with acute conditions is complicated as well (6). Although COVID-19 is principally defined by its respiratory symptoms, it is now clear that the virus can also affect the digestive system (7). Patients with COVID-19 may present with gastroenterology symptoms, such as diarrhea, nausea and/or vomiting, and abdominal pain with no respiratory symptoms (8). As such, COVID-19 may mimic IBD relapse symptoms, adding a diagnostic challenge to this group of patients. Moreover, patients' fear to visit the hospital in addition to the shortage of medical resources may cause diagnosis and treatment delay, consequently leading to treatment failure or even to the need of urgent surgical intervention (e.g., in patients with severe ulcerative colitis or complications). According to our survey, there was a rise, though not significantly, in the number of patients who paid a visit to the emergency room for medical care in Hubei province [1 (0.7%) vs. 3 (2%)].

As for today, the current guidelines from the main medical societies suggests maintaining current medication (e.g., immunosuppressive and biological agents) as a preventive strategy in IBD patients without symptoms suggestive of COVID-19 (5). Whether patients who stopped IBD drugs experienced IBD flares leading to hospitalizations and surgeries needs to be further addressed. According to a recent study by Bezzio et al. (9) which presented the characteristics and outcomes of IBD patients with COVID-19, active disease, old age, and comorbidities were risk factors of a negative outcome of COVID-19, whereas IBD medication was not. Global data from the SECURE-IBD registry (https://covidibd.org/) show that older age and health conditions are the major drivers of more severe COVID-19 and death. Steroid use continues to be the strongest medication-associated risk factor. Other IBD medications including anti-TNF biologics appear to be safe. According to our survey, there was no significant change in use of steroids, thiopurines, and aminosalicylates before and after the pandemic outbreak in both sites. However, there were significantly more patients with delayed biologics infusions in the epicenter compared to the non-epicenter area [60.4% (90/149) vs. 19.1% (62/324), *p* < 0.001], which implies that intravenous infusions in the hospital were affected more in the epicenter than that in the non-epicenter area.

According to the web-survey conducted by ECCO (5), physical contact with other people was feared by about half of respondents (45.1%), and most specialists (73.2%) canceled or rescheduled consultations due to the COVID-19 outbreak. In this way, telemedicine may serve as a perfect solution to counteract these challenges. As demonstrated in our survey, patients turned to telemedicine including hospital-based online clinics and Wechat consultations as an alternative way of seeking medical advice. There was an increased use and need of telemedicine after the COVID-19 outbreak especially in Hubei province, the epicenter area. According to our survey, two-thirds of IBD patients from both sites equally support the notion of increasing telemedicine in future medical care (*p* = 0.39). The rapidly developing technological advances in artificial intelligence and virtual reality provide a solid foundation for delivering the right care to the right patient at the right time. It is time to look beyond the traditional role of telemedicine as a connectivity only tool.

The study may be limited in some way. The major limitation of this study is that the survey returns may have been affected by the disaster conditions within the provinces, especially for severely ill patients, patients with a low education level, and patients with other chronic diseases. Some of these patients may have been hospitalized COVID-19 patients who were not able to answer the survey. This may be a bias that would be difficult to overcome due to the conditions during the pandemic.

In summary, our patient-based survey study in a real-world setting showed that COVID-19 resulted in a great impact on the healthcare of patients with IBD, and that such an impact was more obvious in the epicenter compared to the non-epicenter area of COVID-19. Telemedicine which transcends geography offers a good solution to counteract the challenges in such an unprecedented situation such as COVID-19, and there is support for more widespread adoption of telemedicine among patients.

## Data Availability Statement

The raw data supporting the conclusions of this article will be made available by the authors, without undue reservation.

## Ethics Statement

The studies involving human participants were reviewed and approved by The Ethics Committee of the First Affiliated Hospital of Sun Yat-sen University. Written informed consent from the participants' legal guardian/next of kin was not required to participate in this study in accordance with the national legislation and the institutional requirements.

## Author Contributions

RM and M-HC conceived the study and supervised the overall study. RM, YQ, and Y-FZ wrote the manuscript. L-RZ, J-SH, N-DT, J-YT, S-NL, X-QL, and SG critically revised the manuscript. All authors contributed to the article and approved the submitted version.

## Conflict of Interest

The authors declare that the research was conducted in the absence of any commercial or financial relationships that could be construed as a potential conflict of interest.
