# Supplementary material for: Impact of COVID-19 on the Healthcare of Patients With Inflammatory Bowel Disease: A Comparison Between Epicenter vs. Non-epicenter Areas
Source: Front Med (Lausanne). 2020 Nov 30;7:576891. doi: 10.3389/fmed.2020.576891 (PMC7734214; doi:10.3389/fmed.2020.576891)
Supplement: Supplementary file 1 [file Table_1.DOCX]

**Questionnaire for patients**

[General information]

1. What is your current diagnosis?

A. Cronh’s disease B. Ulcerative colitis C. Others__________(describe please)

1. What is your gender?

A. Male B. Female

1. How old are you?

A. ≤16 years old

B. 16~40 years old (>16 years old, ≤40 years old)

C. 40~65 years old (>40 years old, ≤65 years old)

D. >65 years old

1. What is your residential address? (Detailed to the prefecture-level city please)

_______ Province _________City__________________

1. How long have you been diagnosed with inflammatory bowel disease?

A.≤2 years

B.2~5 years (>2 years，≤5 years)

C.5~10 years (>5 years，≤10 years)

D. >10 years

1. How long has it been since your last visit to hospital? (until 2020.04)

A. ≤3month

B. 3~6month（>3month，≤6month）

C. 6~12month（>6month，≤12month）

D. >12month

[Before the outbreak of COVID-19]

1. Which drug did you use one month before the outbreak of the COVID-19 epidemic (2019.12.25-2020.1.23)? (Multiple choices are available)

A. Glucocorticoid (Medrol, Methylprednisolone, Prednisone, etc.)

B. Imuran (Azathioprine) / Mercaptopurine Tablets

C. Thalidomide Tablets

D. Methotrexate Tablets (Oral)

E. Methotrexate injection (Intramuscular / hypodermic injection)

F. Remicade (Infliximab for injection)

G. Adalimumab

H. Drugs of clinical trials

I. Sulfasalazine

J. Mesalazine (Etiasa, Salofalk, Pentasa etc.)

K. None

L. Others_________ (describe please)

1. How did you get drugs one month before the outbreak of the COVID-19 epidemic (2019.12.25-2020.1.23)? (Multiple choices are available)

A. Prescribed from outpatient clinics in hospital

B. Prescribed from emergency clinics in hospital

C. Purchased in pharmacies

D. Purchased on the Internet platform (JD.com, Taobao, Jianke, etc.)

E. Purchased by relatives

F. Not purchased due to sufficient amount of medicine

G. Others_________ (describe please)

1. How did you get medical treatment one month before the outbreak of COVID-19 epidemic (2019.12.25-2020.1.23)? (Multiple choices are available)

A. Outpatient clinics in hospital

B. Emergency clinics in hospital

C. Internet platform built by hospital (e.g. cloud clinic)

D. Other Internet platforms (e.g. haodf.com)

E. Short Messaging Consultation

F. Call for Consultation

G. Consultation on WeChat

H. Video consultation

I. None

J. Others_________ (describe please)

[After the outbreak of COVID-19]

1. Which drug did you use one month after the outbreak of the COVID-19 epidemic (2020.1.24-2020.2.29)? (Multiple choices are available)

A. Glucocorticoid (Medrol, Methylprednisolone, Prednisone, etc.)

B. Imuran (Azathioprine) / Mercaptopurine Tablets

C. Thalidomide Tablets

D. Methotrexate Tablets (Oral)

E. Methotrexate injection (Intramuscular / hypodermic injection)

F. Remicade (Infliximab for injection)

G. Adalimumab

H. Drugs from clinical trials

I. Sulfasalazine

J. Mesalazine (Etiasa, Salofalk, Pentasa etc.)

K. None

L. Others_________ (describe please)

1. Could you purchase medicine in time one month after the outbreak of the COVID-19 epidemic? (2020.1.24-2020.2.29)?

A. Yes B. No C. Not purchased due to enough medicine

1. How did you get drugs one month after the outbreak of the COVID-19 epidemic in 2020 (2020.1.24-2020.2.29)? (Multiple choices are available)

A. Prescribed from outpatient clinics in hospital

B. Prescribed from emergency clinics in hospital

C. Purchased in pharmacies

D. Purchased on the Internet platform (JD.com, Taobao, Jianke, etc.)

E. Purchased by relatives

F. Not purchased due to sufficient amount of medicine

G. Others_________ (describe please)

1. Did you stop your medication or switch to other treatments due to insufficient medication one month after the outbreak of the COVID-19 epidemic in 2020 (2020.1.24-2020.2.29)?

A. Yes, the withdrawal time was ___ days B. No C. I switched to other treatments

1. Did you remain stable condition one month after the outbreak of the COVID-19 epidemic in 2020 (2020.1.24-2020.2.29)?

A. Yes B. No C. Not sure

1. How did you get medical treatment one month after the outbreak of COVID-19 epidemic in 2020 (2020.1.24-2020.2.29)? (Multiple choices are available)

A. Outpatient clinics in hospital

B. Emergency clinics in hospital

C. Internet platform built by hospital (e.g. cloud clinic)

D. Other Internet platforms (e.g. haodf.com)

E. Short Messaging Consultation

F. Call for Consultation

G. Consultation on WeChat

H. Video consultation

I. None

J. Others_________ (describe please)

1. How many times did you use the Internet platform (WeChat, SMS, cloud clinic, haodf.com, etc.) for consultation one month after the outbreak of the COVID-19 epidemic (2020.1.24-2020.2.29)?

A. ≤2 times B. 2-5 times (> 2times, ≤5times) C.> 5 times

1. Were your inspections related to inflammatory bowel disease delayed one month after the outbreak of the COVID-19 epidemic (2020.1.24-2020.2.29)?

A. Yes, they have been done after the delay (how many__ days delayed)

B. Yes, they have not been done after the delay

C. No, they have not been delayed

D. No scheduled inspection

1. Was your treatment of biological agents (e.g. Infliximab, Adalimumab and drugs of clinical trials) delayed one month after the outbreak of the COVID-19 epidemic (2020.1.24-2020.2.29)?

A. I didn’t take biological agent treatments

B. No (the name of the biological agent is ______)

C. Yes, the delayed biological agent has been injected (the delayed time is __ days, the name of the biological agent is_______)

D. Yes, the delayed biological agent has not been injected (The name of the biological agent is ______)

1. Were your surgeries related to inflammatory bowel disease delayed one month after the outbreak of the COVID-19 epidemic (2020.1.24-2020.2.29)?

A. Yes, they have been done after the delay (how many__ days delayed)

B. Yes, they have not been done after the delay

C. No, they have not been delayed

D. No planed surgery

[Expectations after the COVID-19 epidemic]

1. Which way of consultation would you prefer after the epidemic?

A. Face-to-face consultation in hospital outpatient clinics

B. Internet platform built by hospital (e.g. cloud clinic)

C. Other internet platforms (e.g. haodf.com)

D. Short Messaging Consultation

E. Call for Consultation

F. Consultation on WeChat

G. Video consultation

H. Others _________ (describe please)

1. Would you like online ways provided for diagnosis and treatment after the epidemic?

A. Yes B. No C. Not sure

1. In your opinion, which aspects of the existing online platforms need to be improved to meet future needs? (Multiple choices are available)?

A. Improvement in internet platform technology

B. More doctors providing diagnosis and treatment services on the Internet

C. Subsequent medical-insurance-related measures for online medical treatment

D. Improvement in relevant inspections and drug distribution system

E. _________ (describe please)

【Respiratory symptoms】

1. Did you have a fever during the epidemic?

A. Yes, in 1 month before the outbreak (2019.12.25-2020.1.23)

B. Yes, in 1 month after the outbreak (2020.1.24-2020.2.29)

C. Yes, in 1 month before and after the outbreak

D. No

1. Did you have a cough during the epidemic?

A. Yes, in 1 month before the outbreak (2019.12.25-2020.1.23)

B. Yes, in 1 month after the outbreak (2020.1.24-2020.2.29)

C. Yes, in 1 month before and after the outbreak

D. No

1. Did you have a running nose during the epidemic?

A. Yes, in 1 month before the outbreak (2019.12.25-2020.1.23)

B. Yes, in 1 month after the outbreak (2020.1.24-2020.2.29)

C. Yes, in 1 month before and after the outbreak

D. No

1. Did you have a chest tightness during the epidemic?

A. Yes, in 1 month before the outbreak (2019.12.25-2020.1.23)

B. Yes, in 1 month after the outbreak (2020.1.24-2020.2.29)

C. Yes, in 1 month before and after the outbreak

D. No

1. Did you have other respiratory symptoms during the epidemic?

A. Yes, in 1 month before the outbreak (2019.12.25-2020.1.23), and it was______ (describe please)

B. Yes, in 1 month after the outbreak (2020.1.24-2020.2.29), and it was______ (describe please)

C. Yes, in 1 month before and after the outbreak, and it was______ (describe please)

D. No

1. During the epidemic, have you ever visited the fever clinic for consultation?

A. Yes, in 1 month before the outbreak (2019.12.25-2020.1.23)

B. Yes, in 1 month after the outbreak (2020.1.24-2020.2.29)

C. Yes, in 1 month before and after the outbreak

D. No

1. Have you taken any tests related to Covid-19 during the epidemic?

A. Throat swab specimens for detection of SARS-CoV-2 Ribonucleic Acid (RNA) was taken and it was negative.

B. Throat swab specimens for detection of SARS-CoV-2 Ribonucleic Acid (RNA) was taken and it was positive.

C. Computed tomography (CT) of the chest was taken and it was negative.

D. Computed tomography (CT) of the chest was taken and it was positive.

E. Coronavirus antibody detection (blood test) was negative.

F. Coronavirus antibody detection (blood test) was positive.

G. I haven’t taken any tests.

1. Were there any of your family members diagnosed with Coronavirus Disease 2019 (COVID-19) during the epidemic?

A. Yes B. No

1. Did you have a history of close contact with COVID-19 patients?

A. Yes B. No
